# Supramodal Enhancement of Auditory Perceptual and Cognitive Learning by Video Game Playing

**DOI:** 10.3389/fpsyg.2017.01086

**Published:** 2017-06-28

**Authors:** Yu-Xuan Zhang, Ding-Lan Tang, David R. Moore, Sygal Amitay

**Affiliations:** ^1^Medical Research Council Institute of Hearing Research, School of Medicine, University of NottinghamNottingham, United Kingdom; ^2^State Key Laboratory of Cognitive Neuroscience and Learning and IDG/McGovern Institute for Brain Research, Beijing Normal UniversityBeijing, China; ^3^Communication Sciences Research Center, Cincinnati Children’s Hospital Medical Center, CincinnatiOH, United States

**Keywords:** perceptual training, auditory learning, video game, working memory, tone frequency discrimination, tone n-back

## Abstract

Medical rehabilitation involving behavioral training can produce highly successful outcomes, but those successes are obtained at the cost of long periods of often tedious training, reducing compliance. By contrast, arcade-style video games can be entertaining and highly motivating. We examine here the impact of video game play on contiguous perceptual training. We alternated several periods of auditory pure-tone frequency discrimination (FD) with the popular spatial visual-motor game Tetris played in silence. Tetris play alone did not produce any auditory or cognitive benefits. However, when alternated with FD training it enhanced learning of FD and auditory working memory. The learning-enhancing effects of Tetris play cannot be explained simply by the visual-spatial training involved, as the effects were gone when Tetris play was replaced with another visual-spatial task using Tetris-like stimuli but not incorporated into a game environment. The results indicate that game play enhances learning and transfer of the contiguous auditory experiences, pointing to a promising approach for increasing the efficiency and applicability of rehabilitative training.

## Introduction

Rehabilitative training has long been used to improve perceptual and cognitive performance in normal (Mahncke et al., 2006; Takeuchi et al., 2010) as well as clinical (learning impairement, Merzenich et al., 1996; e.g., amblyopia, Maurer and Hensch, 2012; stroke, Taub, 2012) populations. However, rehabilitative training, typically consisting of many hours of repetitive practice, is often too effortful and tedious for the intended users (Levi and Li, 2009). In contrast, computer games, which can be entertaining and easily modified to suit different users, have become a ubiquitous part of modern life. Playing fast action video games (with audio effects) has been shown to improve a wide range of visual perception and attention skills (Green and Bavelier, 2003, 2012). Auditory benefits of game play, on the other hand, have typically been studied using laboratory designed auditory games (Honda et al., 2007; Whitton et al., 2014; Wise et al., 2015) rather than entertaining video games. In one notable exception, we previously reported that playing Tetris, a popular arcade type video game involving fast visual-motor control, improved auditory perception (Amitay et al., 2006). This result raised the possibility that existing video games may be utilized for auditory rehabilitation or enhancement of auditory skills. Here we re-examine the supramodal effect of video game play on auditory learning.

In our previous report (Amitay et al., 2006), after an hour of Tetris play, either in silence or accompanied by passive presentation of tones, players improved on tone frequency discrimination (FD), and the FD improvement was correlated with Tetris learning. There are at least three possible mechanisms for Tetris play to improve auditory perception. The first account is that auditory learning of Tetris players may reflect general behavioral improvements induced by familiarization with the training procedures and environment. This is often referred to as procedural learning. It occurs at the beginning of training and saturates rapidly (Hawkey et al., 2004; Ortiz and Wright, 2009). In the previous one-session training experiment (Amitay et al., 2006), FD testing prior to training was very brief (∼2 min). Procedural learning could therefore have continued into the training session. Second, playing Tetris may improve some supra-modal cognitive functions such as attention or working memory (WM), which in turn improves auditory perception. For example, we have shown that WM training can improve FD performance (Zhang et al., 2016). A third possibility is that game play interacts across time and modality with auditory experiences, enhancing rather than generating auditory learning. Supporting this possibility, within-modal learning enhancement has been reported for contiguous auditory (Wright et al., 2010) and visual (Szpiro et al., 2014) stimulus exposure.

**FIGURE 1:**
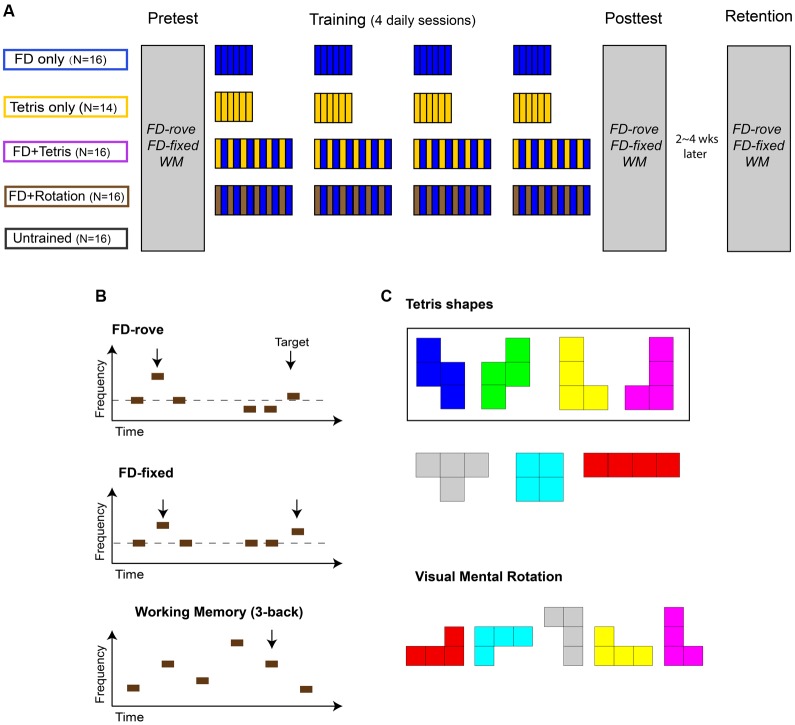
Training paradigm and tasks. **(A)** Training paradigm. Four training groups (FD only, Tetris only, FD + Tetris, and FD + Rotation), together with a fifth, no-training control group, were tested before (Pretest) and after (Posttest) 4 daily training sessions on the same three tasks. **(B)** Pre- and Post-test tasks. Top row: Frequency discrimination with a standard tone frequency roving from trial to trial between 0.9 and 1.1 kHz (FD-rove). Each trial consisted of three tones, two identical and one higher in frequency (target). Participants were to indicate the target tone. Middle row: Frequency discrimination with a fixed standard frequency of 1 kHz (FD-fixed). Bottom row: auditory working memory (WM) measured with Tone 3-back. Each trial consisted of 43 tones with salient frequency differences. Participants were to press a button if a tone were the same as 3 positions back (target). **(C)** Visual mental rotation task. Top: Four of the seven Tetris shapes (the asymmetric ones in the rectangle) and all seven colors were used. Bottom: A sample stimulus of five shapes. Participants were to indicate whether all shapes were the same regardless of angle and color. See text for further details.

Here, we tested the effect of Tetris play on auditory learning using a multi-session training paradigm designed to distinguish these three possible mechanisms (**Figure 1**). We trained FD with a roved standard frequency (FD-rove; **Figure 1B**), a task that we have previously shown to produce learning over multiple sessions (Amitay et al., 2005) and transfer to auditory WM (Zhang et al., 2016). After a 90-min pretest, four training groups practiced for four daily sessions on either FD, Tetris in silence, FD interleaved with Tetris, or FD interleaved with another mental rotation task. All groups were compared with an untrained control group for evaluation of training. The procedural learning hypothesis predicts no Tetris effect because the pretest session was long enough to saturate procedural learning (Hawkey et al., 2004). The cognitive learning hypothesis predicts auditory benefits for practicing Tetris only. In contrast, the learning enhancement hypothesis predicts auditory benefits for Tetris interleaved with FD, but not for Tetris only. Finally, if the Tetris effect arises from the visual spatial skills involved in Tetris play, a similar effect should be observed with the mental rotation task.

## Materials and Methods

### Participants

Seventy eight paid participants aged 18–35 (43 females) were recruited from the University of Nottingham campus and neighboring communities. All participants had normal hearing (<=20 dB HL on tone audiogram between 0.5 and 4 kHz) and gave informed written consent. The research protocol was approved by the Nottingham University Hospitals Research Ethics Committee.

### Experimental Design

The experiment consisted of a Pretest session, four training sessions, and a Posttest session on consecutive days except weekends (**Figure 1A**), similar to the training paradigm that has been previously shown to produce learning on FD-rove (Amitay et al., 2005). A retention-test session was given 2–4 weeks after the Posttest. Following Pretest, participants were assigned randomly into four training groups and one no-contact control group.

In Pre-, Post- and Retention tests participants performed probe tasks of tone FD, with roving and fixed standards. Auditory working memory for frequency was also tested (see below for task description). Task order was randomized across participants but fixed within individuals. In each training session, the training groups practiced FD with a roving standard (FD only; *n* = 16) for 900 trials (∼35 min), Tetris (Tetris only; *n* = 14) played for ∼35 min (downloaded for free from http://sivut.koti.soon.fi/sodacan; as used in Amitay et al., 2006), FD alternated approximately every 6 min with silent play of Tetris (FD + Tetris; *n* = 16) or with visual mental rotation (FD + Rotation, *n* = 16; **Figure 1C**). The Control group (*n* = 16) did not receive any training, but had the same number of days between pre- and post-test sessions as the training groups. Seventy-one participants returned for the retention test.

### Task and Stimuli

#### Frequency Discrimination (FD) Tasks

For FD tasks (**Figure 1B**, top rows), each trial consisted of three sequentially presented tones, two of which were identical (standards) and the third had a higher frequency (the target). The target tone’s temporal position was randomized across trials. Participants were instructed to indicate the position of the target by pressing a button. Feedback was provided visually after each trial. All tones were 100 ms long, including 10-ms rise-fall ramps, presented at 75 dB SPL with an interstimulus interval (ISI) of 300 ms. In the fixed-standard task (FD-fixed), the standard frequency was always 1 kHz. In the roving-standard task (FD-rove), the standard frequency varied randomly from trial to trial between 0.9 and 1.1 kHz in 50 Hz steps.

For FD testing and training, the frequency difference between the target and standard (ΔF, expressed as percentage of the standard) was adaptively varied in blocks of 50 trials. In each block, ΔF started at 50%, and was divided by 2 after every correct response until the direction of change switched from decreasing to increasing. Thereafter, the adaptive rule switched to 3-down 1-up, where ΔF was divided by 1.41 after three consecutive correct responses or multiplied by the same factor after one incorrect response, to estimate FD threshold at 79% correct point on the psychometric function (Levitt, 1971). The roving-standard task was used for training because with this amount of roving, performance continued to improve through multiple sessions of practice (Amitay et al., 2005) and learning transferred to WM (Zhang et al., 2016). Increasing the roving range could disrupt learning in some participants (Amitay et al., 2005). Two blocks of each FD task were presented in each test session, and 18 blocks of FD-rove were presented in each training session. Performance of each session was evaluated by the average threshold obtained in that session. All statistical analyses were conducted on log-transformed FD data, resulting in normal distributions (Shapiro–Wilk test, *p* > 0.16).

#### Working Memory (Tone 3-Back) Task

Auditory WM was measured using a 3-back task with tonal stimuli (Zhang et al., 2016). The task (**Figure 1B**, bottom row) consisted of 43 sequential 100-ms tones with an ISI of 2,400 ms. Participants were instructed to press a button if the current tone matched the tone 3 positions back (a target). Twelve of the last 40 tones, randomly selected for each sequence, were targets. No responses were required for non-targets. At the end of each sequence, performance feedback (percent correct) was visually provided. All tones were presented at 60 dB SPL. Each sequence contained eight frequencies drawn between 1,080 and 4,022 Hz and separated by at least one equivalent rectangular bandwidth (Moore, 2003), so that the frequencies were clearly distinguishable from each other. Performing this task requires maintenance of at least 4 tones in working memory and constant updating of that memory.

Before starting the 3-back task, participants completed one or two 20-tone sequences of a 2-back version of the task to get familiarized. Two sequences of the 3-back task were administered in each testing session. A sensitivity index (d’) was calculated from hit and false-alarm rates for each sequence. Average d’ of the two sequences was used for session performance.

#### Visual Mental Rotation Task

The visual mental rotation task (**Figure 1C**) was modified after Sims and Mayer (2002). It was designed to reflect the visual spatial skills involved in the game Tetris. The four asymmetric of the seven Tetris shapes were used as stimuli (two pairs of mirror-image stimuli). On each trial, a set of stimuli, randomly selected from the asymmetric shapes, was presented against an invisible 5 by 5 grid (140 by 140 pixels each cell) at the center of a white screen of 1024 by 768 pixels. The grid was filled from top, with one shape (105 by 70 pixels) placed in the center of a cell. The angle of each shape was randomized with a step of 90° (rotated 0, 90, 180, or 270°). Each shape was presented in one of the seven colors used in the Tetris game, randomly selected at each trial. In contrast to the usual Tetris game, in the mental rotation task there was no match between colors and shapes, so that shape had to be used to solve the task. On half the trials, randomly chosen, all shapes were the same. On the other half of the trials, a single mirror-image shape was embedded in the array (the target). The participant’s task was to judge whether such a target was present as accurately and as quickly as possible by pressing a button. The same visual feedback as in FD tasks was provided after each response. The set size (number of shapes in a display) was randomly selected from 5, 10, and 15 across trials. A pilot study indicated that compared to a fixed set size, the randomization method promoted the challenge and learning of the mental rotation task. Both performance accuracy and reaction time were recorded.

### Statistical Analyses

**FIGURE 2:**
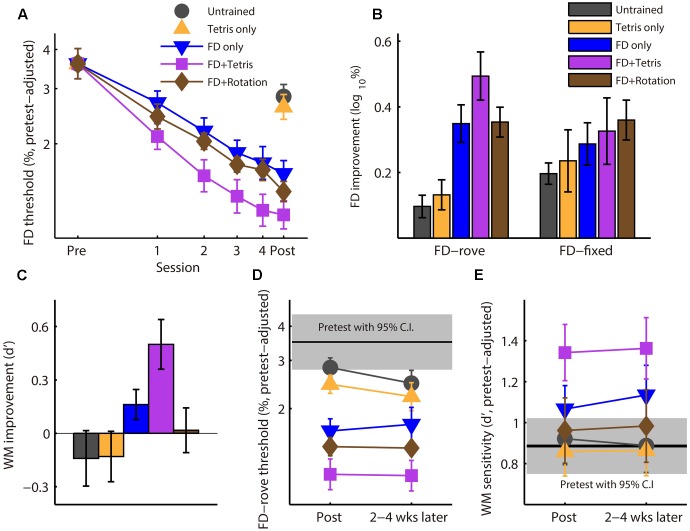
Effect of Tetris play on learning, transfer, and retention of auditory discrimination training. **(A)** Learning during training. FD thresholds on training and posttest sessions were adjusted to account for variation in the pretest score. **(B)** Improvement from the pretest to the posttest for FD with a roving (FD-rove, trained) and a fixed (FD-fixed, untrained) standard. **(C)** WM improvement, presented as increase in d’ from the pretest to the posttest. **(D)** Retention of FD-rove improvement 2–4 weeks after the posttest. **(E)** Retention of WM improvement. Error bars in all panels represent SEMs.

For FD tasks, thresholds in percent of standard frequency were log-transformed for data analyses (Zhang et al., 2016). For Tone 3-back, performance was measured by d’, calculated as Z(hit rate) – Z(false alarm rate), where Z is the inverse cumulative Gaussian distribution. Pretest performance for each task was compared among the five participant groups with a one-way ANOVA. Learning of the three FD training groups was compared using group by session ANOVAs with repeated measures on session. Transfer to untrained tasks between the pre- and posttest was examined using group by test ANOVAs with repeated measures on test. Similar ANOVAs were conducted on the posttest and retention test to examine retention of learning. FD thresholds were highly variable across individuals. To better illustrate performance change over time, in **Figure 2**, FD thresholds during training, posttest, and retention were adjusted to account for individual differences on the pretest.

## Results

### Tetris But Not Visual Rotation Enhanced FD Learning

Before training, there was no group difference in FD performance with either a roving (FD-rove, the training task; ANOVA group effect: *F*_4,73_ = 1.21, *p* = 0.32) or a fixed (FD-fixed, untrained; *F*_4,73_ = 0.33, *p* = 0.86) standard frequency. During training, FD-rove threshold improved (decreased) significantly for all three FD-rove training groups (**Figure 2A**; repeated-measure ANOVA, main effect of session: *F*_5,225_ = 80.8, *p* < 0.001, $\eta_{p}^{2}$ = 0.62), but the improvement differed among groups (group by session interaction: *F*_10,225_ = 2.9, *p* = 0.002, $\eta_{p}^{2}$ = 0.11). Planned between-group comparisons revealed that the FD + Tetris group improved more than the FD only group (group by session interaction: *F*_5,150_ = 2.9, *p* = 0.002, $\eta_{p}^{2}$ = 0.11) and the FD + Rotation group (*F*_5,155_ = 3.91, *p* = 0.002, $\eta_{p}^{2}$ = 0.11), but the FD + Rotation group did not differ from the FD only group (*F*_5,150_ = 0.27, *p* = 0.93, $\eta_{p}^{2}$ = 0.009). The FD + Rotation group did improve on the visual mental rotation task, as reaction time decreased over training sessions (ANOVA, effect of session: *F*_3,42_ = 46.3, *p* < 0.001, $\eta_{p}^{2}$ = 0.77) while accuracy remained constant through training at a close-to-ceiling level (>90%; effect of training session: *F*_3,42_ = 0.34, *p* = 0.80). The training results indicate that silent Tetris play intermixed with auditory training enhanced FD learning. This training could not be accounted for by the visual-spatial training involved in game play.

Between the pre- and posttest, performance on the trained, FD-rove task changed differently among the five groups (**Figure 2B**; group by test interaction: *F*_4,73_ = 11.6, *p* < 0.001, $\eta_{p}^{2}$ = 0.39). Planned between-group comparisons revealed that, while all of the three FD-rove training groups improved more than untrained controls (group by test interaction, FD + Tetris: *F*_1,30_ = 28.5, *p* < 0.001, $\eta_{p}^{2}$ = 0.49; FD only: *F*_1,30_ = 14.3, *p* = 0.001, $\eta_{p}^{2}$ = 0.32; FD + Rotation: *F*_1,29_ = 20.4, *p* < 0.001, $\eta_{p}^{2}$ = 0.41), the Tetris only group did not (*F*_1,28_ = 0.40, *p* = 0.54). Further, the FD + Tetris group improved FD-rove more than the FD only group (*F*_1,30_ = 4.41, *p* = 0.044, $\eta_{p}^{2}$ = 0.13) and the FD + Rotation group (*F*_1,30_ = 4.83, *p* = 0.036, $\eta_{p}^{2}$ = 0.14). The learning-enhancing effect of Tetris play (**Figure 2A**) must therefore have resulted from an interaction between game play and auditory training. All groups improved on the untrained FD-fixed task (**Figure 2B**; effect of test: *F*_1,73_ = 67.2, *p* < 0.001, $\eta_{p}^{2}$ = 0.48), but there was no difference between groups (group by test interaction: *F*_4,73_ = 0.92, *p* = 0.46), indicating that learning on the FD-fixed task saturated by end of the pretest. As the two FD tasks were similar in procedure and test environment, it is unlikely that procedural learning should continue with FD-rove but not with FD-fixed. Thus, the additional learning induced by FD training with a roving standard, compared with untrained controls, should be indicative of perceptual learning.

### Tetris-Enhanced Transfer of FD Learning to WM

We also tested whether Tetris play influenced transfer of FD learning to Tone 3-back, an auditory WM task (**Figure 1B**). Before training, there was no group difference in Tone 3-back performance (*F*_4,72_ = 2.0, *p* = 0.10). The five groups changed differently from the pretest to the posttest (**Figure 2C**; *F*_4,70_ = 4.2, *p* = 0.004, $\eta_{p}^{2}$ = 0.19). Planned group comparisons showed that the Tetris only group did not differ from the Control group (*F*_1,27_ = 0.002, *p* = 0.96). Neither did the FD only group (*F*_1,30_ = 2.92, *p* = 0.098) or the FD + Rotation group (*F*_1,28_ = 0.60, *p* = 0.45). The FD + Tetris group, however, improved significantly more than the Control group (*F*_1,30_ = 9.37, *p* = 0.005, $\eta_{p}^{2}$ = 0.24). Moreover, the FD + Tetris group improved WM more than the FD only group (*F*_1,30_ = 4.29, *p* = 0.047, $\eta_{p}^{2}$ = 0.13) and the FD + Rotation group (*F*_1,28_ = 6.44, *p* = 0.017, $\eta_{p}^{2}$ = 0.19). Thus, intermixing Tetris play with FD training promoted transfer to WM. Similar to the learning-enhancing effect, this effect was also unaccounted for by Tetris play alone or visual rotation training.

### Tetris Effects Were Retained

Most of the participants (*n* = 71; 13–15 per group) returned 2–4 weeks after the posttest for a ‘retention’ test. There was no significant difference between the posttest and retention test for the trained FD-rove task (**Figure 2D**; repeated measure ANOVA, main effect of test: *F*_1,65_ = 0.91, *p* = 0.34, $\eta_{p}^{2}$ = 0.014; test by group interaction: *F*_4,65_ = 0.83, *p* = 0.51, $\eta_{p}^{2}$ = 0.048) or the untrained FD-fixed task (data not shown; main effect of test: *F*_1,65_ = 2.09, *p* = 0.15, $\eta_{p}^{2}$ = 0.031; test by group interaction: *F*_4,65_ = 0.56, *p* = 0.69, $\eta_{p}^{2}$ = 0.033). Improvement in WM sensitivity was also retained (**Figure 2E**; main effect of test: *F*_1,59_ = 0.02, *p* = 0.89, $\eta_{p}^{2}$ < 0.001; test by group interaction: *F*_4,59_ = 0.14, *p* = 0.97, $\eta_{p}^{2}$ = 0.010). Planned between-group comparisons revealed that the advantage of the FD + Tetris group over the FD only group was retained for the trained FD-rove task (main effect of group: *F*_1,24_ = 4.56, *p* = 0.043, $\eta_{p}^{2}$ = 0.16) and WM (*F*_1,19_ = 4.56, *p* = 0.046, $\eta_{p}^{2}$ = 0.19), but without further learning (main effect of test, FD-rove: *F*_1,65_ = 0.91, *p* = 0.34, $\eta_{p}^{2}$ = 0.014; WM: *F*_1,59_ = 0.020, *p* = 0.89, $\eta_{p}^{2}$ < 0.001).

## Discussion

The results demonstrate that game play enhances perceptual learning of contiguous auditory experiences. Confirming and extending our previous report of auditory benefits of Tetris play in brief, single-session training (Amitay et al., 2006), we showed in a multi-session training paradigm that Tetris play in isolation produced no benefits for an FD-rove task. This indicated that the previously observed auditory benefits did not result from improved supra-modal cognitive function. However, Tetris play did enhance this form of FD learning and its transfer to WM when mixed with auditory training. The learning enhancement cannot be attributed to procedural learning or familiarization with the testing environment, as the pretest session was long enough to saturate learning on the easier FD-fixed task. Nor can the enhancement be attributed to the extended training duration or visual spatial training brought by Tetris play, as the enhancement was gone when Tetris was replaced by a visual mental rotation task with similar stimuli.

The supramodal nature of the observed learning enhancement challenges existing theories of perceptual learning. In training, mixing two perceptual tasks has been shown to produce transfer between the tasks both in vision (Zhang et al., 2010) and in audition (Wright et al., 2010). It was suggested that this intramodal transfer results from the application of neural computation (Zhang et al., 2010) or resources (Wright et al., 2010) required for learning the target task to sensory inputs of the non-target task. In both views, the role of the non-target task is to provide exposure to the stimulus feature to be learned. However, in the current study, no such sensory stimulation for auditory learning was provided by Tetris, which was played in silence. Another example of enhanced learning through a non-target task is an increase of sensitivity to background stimuli while performing a foreground task (Watanabe et al., 2001). Seitz and Watanabe (2005) proposed that diffuse reinforcement signals produced by a foreground (target) task enhanced processing of concurrent background (non-target) stimuli. However, the task-irrelevant learning occurred only when the background stimuli were subliminal and coincident with the reinforced targets of the foreground task (Choi and Watanabe, 2009). In the current study, learning enhancement violated both of these requirements.

The game training literature provides no clue that we can find to the nature of the supramodal enhancement of auditory learning and transfer by Tetris play. Though playing action games improves visual perception and attention, Tetris training alone does not improve unrelated perceptual or cognitive tasks (Sims and Mayer, 2002; Green and Bavelier, 2003). Consistently, in the current study, we found no evidence that playing Tetris had any auditory benefits beyond the pretest. Indeed, auditory benefits of game play have only been reported for games specially designed to emphasize the role of auditory stimuli (Honda et al., 2007; Whitton et al., 2014). In contrast, the game-enhanced perceptual and cognitive learning observed here required no auditory stimuli during game play and must therefore involve different mechanisms from those induced by game play alone.

The results indicate that auditory training and transfer was enhanced by some factors that were provided by Tetris play but not by the visual-spatial stimulation involved in the game. One candidate of such learning-enhancing factors is reward signals. Playing video games, even the arcade-style ones, can lead to release of reward signals such as dopamine (Koepp et al., 1998), which promotes synaptic plasticity (Buchanan et al., 2010; Giessel and Sabatini, 2010) and contributes to experience-dependent learning (for review, see Harley, 2004; Glimcher, 2011). Conventional behavioral training, like the auditory discrimination training examined in the current study, involves extensive repetition that can be far from rewarding or motivating. Compared with game play, such training may be accompanied by a low level of reward signals, making it sensitive to the learning-boosting effects brought by an increase of reward signals. For example, coupling auditory stimuli with electrical stimulation of the dopamine network has been shown to enable and enhance cortical reorganization in primary as well as association cortices (Bao et al., 2001). Game play could function as a behavioral stimulator of the reward system, promoting learning of contiguous sensory experiences in a similar way as stimulating the dopamine network enhances reorganization of cortical responses to accompanied auditory stimuli (Bao et al., 2001).

Note that the learning-enhancing effect of game play requires no temporal coupling of the rewarding event (game play) with the target training (auditory discrimination), unlike the aforementioned task-irrelevant learning, which occurs only to the subliminal background stimuli that are synchronous with the presumably reward-producing target (Watanabe et al., 2001). As proposed for the task-irrelevant learning, in conventional training reward signals are considered to play a reinforcing role, in that their fluctuations in level allowed trial-by-trial feedback to modulate behavior (Watanabe et al., 2001). In contrast, by mixing game play with training, we seek to increase systematically the level of reward signals throughout training. The reward signals produced by game play may not be informative in improving performance on the perceptual task on a trial-by-trial level. Rather, reward signals may purportedly gate neural plasticity by encoding the ecological significance of the experiences (Gee and Oertner, 2016). Consistent with the plasticity modulating role, dopamine activity has been shown to promote subsequent associative learning (Popescu et al., 2016) and consolidation of associative memory (Schicknick et al., 2012) in animals. In humans, orally taken dopamine precursors, which would increase plasma dopamine level for tens of minutes to hours, have been reported to enhance motor (Floel et al., 2005) and word (Knecht et al., 2004) learning. In this view, the proximity of game play and perceptual training could enhance the significance, and hence the induced plasticity, of the training. Consequently, the benefits may not depend on task or stimulus configuration, making training-while-playing widely and conveniently applicable in educational and rehabilitative settings.

## Ethics Statement

This study was carried out in accordance with the recommendations of the Nottingham University Hospitals Research Ethics Committee with written informed consent from all subjects.

## Author Contributions

Y-XZ and SA conceived and designed the study. Y-XZ performed the experiments, Y-XZ and D-LT analyzed the data, and prepared the manuscript with contributions from SA and DM.

## Conflict of Interest Statement

The authors declare that the research was conducted in the absence of any commercial or financial relationships that could be construed as a potential conflict of interest. The reviewer SA and handling Editor declared their shared affiliation, and the handling Editor states that the process nevertheless met the standards of a fair and objective review.
